# The Value of a Laugh

**Published:** 1912-05

**Authors:** 


### The Value of a Laugh.

"Laugh and grow fat" has physiological truth in it. As laughter, with its accompanying mental cheer and massage to the abdominal organs, increases the digestive power, so the assimilative function of the blood is augmented, and more flesh is made. The quality of the food taken, its preparation in the mouth, and the extent to which the food was needed, determine whether this shall be good, healthy flesh or an accumulation of impurities for disease germs to feed upon.

Laugh and grow fat, and, as you grow fat, you will laugh still more.

Jolly people are one of the greatest boons to humanity. They are their own excuse for living. Who is more popular, more heartily welcomed everywhere than a jolly, happy person? What is more delightful and refreshing than reading a really funny book?

So laugh, and the world will laugh with you, and be a much happier, healthier world in consequence.—*Good Health.*

---

When the abdomen is opened to discover the sigmoid, if it is not found at once, search should be made toward the median line.—*American Journal of Surgery.*

